# Topographic anatomy and anatomic morphometry of abdominal aorta and renal artery versus atherosclerotic plaque formation: apples and pears?

**DOI:** 10.1590/1806-9282.20252164

**Published:** 2026-07-10

**Authors:** Arif Keskin, Ilker Sengul, Demet Sengul, Tayfun Aygun, Elif Karakılıc, Alptekin Tosun

**Affiliations:** 1 Giresun University, Faculty of Medicine, Department of Anatomy – Giresun, Turkey.; 2 Giresun University, Faculty of Medicine, Division of Endocrine Surgery – Giresun, Turkey.; 3 Giresun University, Faculty of Medicine, Department of General Surgery – Giresun, Turkey.; 4 Giresun University, Faculty of Medicine, Department of Pathology – Giresun, Turkey.; 5 Giresun University, Faculty of Medicine, Department of Radiology – Giresun, Turkey.; 6 Trabzon University, Faculty of Medicine, Department of Radiology – Giresun, Turkey.

**Keywords:** Anatomy, Morphometry, Abdominal aorta, Renal artery, Plaque

## Abstract

**OBJECTIVE:**

The aim of this retrospective study was to evaluate the relationship between renal artery morphometry and renal artery plaque formation and location using multislice computed tomography angiography images.

**METHODS:**

The study was conducted retrospectively using multislice computed tomography angiography data from a cohort of 130 patients. Specific measurements included abdominal aortic diameter, contrast material density, right and left renal artery diameters, lengths, angles of aortic separation, and their vertebral levels. Plaque locations in the renal artery and abdominal aorta were recorded. The statistical analysis utilized independent samples t-tests, analysis of variance, and Pearson’s correlation.

**RESULTS:**

A moderate positive correlation (r=0.308, p<0.01) was found between plaque formation and abdominal aortic diameter. An abdominal aortic diameter of ≥20.5 mm was determined as a significant risk threshold for plaque formation in the renal artery (sensitivity: 0.69, specificity: 0.68). The mean arterial length with plaque was significantly longer than that without plaque (difference: 6.97 mm, p=0.029), a relationship that was particularly evident in the right renal artery. In women, a significant association was observed between plaque formation and increased renal artery diameter (difference: 2.10±0.67 mm, p=0.017) and increased renal artery separation angle (difference: 27.44±10.04°, p=0.042). In renal arteries with plaque, both abdominal aorta and renal artery density values were lower than those without plaque (p<0.01).

**CONCLUSION:**

Our findings suggest that the size of the abdominal aorta diameter and the length of the renal artery are important anatomical factors contributing to plaque formation in the renal artery.

## INTRODUCTION

Atherosclerotic renal artery stenosis (RAS) is a leading cause of secondary hypertension. It can lead to refractory hypertension, progressive decline in renal function, and cardiac syndromes (pulmonary edema, acute coronary syndromes) despite medical therapy^1,2^. Studies have shown that patients with RAS are more likely to experience acute coronary syndromes and acute stroke than those without RAS^3^. Although RAS is predominantly caused by atherosclerosis (~90%), data on its prevalence vary, underscoring the importance of the screened population^4^. Individuals are advised medical management, including risk factor control, lipid-lowering medications, renin–angiotensin system antagonists, and antiplatelet therapy. Renal artery plaques (RAPs) are a common consequence of atherosclerosis and may influence the formation of RAS^5^. Although atherosclerosis is considered the most critical risk factor for plaque formation, factors such as age, male gender, body mass index, smoking, hypertension, diabetes, carotid artery intima-media thickness, and aortic and renal artery (RA) calcium scores have been accepted as noninvasive markers of subclinical atherosclerosis and plaque formation^6,7^.

To date, research on RAP has primarily focused on its relationship with subclinical diseases, risk factors, and the progression of systemic atherosclerosis^8,9,10^. However, there are limited studies on the relationship between RAP and anatomical factors^11^. The aim of our study was to determine RA morphometry using multislice computed tomography (MSCT) images and to examine the relationship between RA structure and plaque formation and localization.

## METHODS

### Study design and setting

The study was approved by the Local Scientific Research Ethics Committee. All phases of the study were conducted in accordance with the Declaration of Helsinki. Patient data recorded in our archives were retrospectively reviewed. The patients aged 18 or older who underwent MSCT angiography for any reason were included in the study. Those with a history of previous renal revascularization, renal transplantation, fibro-muscular dysplasia, known congenital RA anomaly, or unclear MSCT images/motion artifacts were excluded from the study. The patient demographic data (age and gender) were obtained from the hospital automation system.

### Data collection and patient management

**Figure 1:**
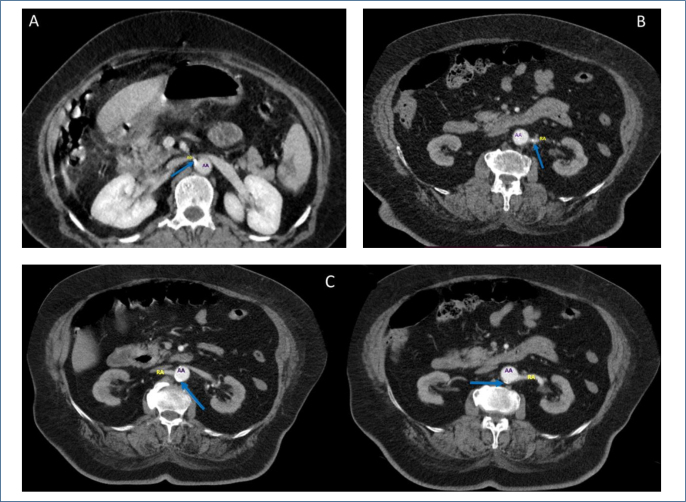
The plaque localization in the renal artery and abdominal aorta. (A) Only in the right renal artery, absent from abdominal aorta; (B) only in the left renal artery, absent from abdominal aorta; (C) only in abdominal aorta, absent from renal arterys. The blue arrows indicate the plaque formations. AA: abdominal aorta; RA: renal artery.

The level of separation of the right and left RAs from the abdominal aorta (AA) was determined according to the level of the vertebral column. The diameters of both RAs at the level of separation from the AA and at the point of bifurcation were measured, and the length between the origin and the point of bifurcation of both RAs was measured. The angles of separation of both arteries from the AA were noted. The density of the opaque material was calculated separately from the aorta at the level of separation of the RA from the AA and from the origin of the RA. Plaque locations were classified as: plaque present only in the RA (absent from the AA), plaque present only in the AA (absent from the RA), or plaque present in both the AA and RA (Figure 1). The separation levels, angles, and length were measured in the axial plane; vessel diameters were measured in both axial and coronal planes. All the computed tomography (CT) scans were performed using a 128-detector CT system (Revolution Evo 128, GE Healthcare, Milwaukee, WI, USA). CT angiography protocols were used for all patients (120 kVp; 100 effective mAs; 0.6 s rotation time; 0.10 mm collimation; 0.984 pitch factor; 1.25 mm helical thickness; 40 mm detector coverage; 65.62 mm/s coverage speed). Morphometric measurements were performed independently by two radiologists. The average of the measurements from both observers was calculated and used in the analyses.

### Statistical analysis

The data were analyzed using IBM SPSS V25 (Statistical Package for the Social Sciences). Normality of distribution was assessed using the Shapiro-Wilk test. An independent samples t-test was employed for between-group comparisons, while a one-way analysis of variance (ANOVA) with a Tukey post hoc test was used for multiple-group comparisons. The Pearson correlation analysis determined relationships between quantitative variables. Univariate logistic regression analysis identified anatomical predictors of RA plaque formation. The receiver operating characteristic (ROC) analysis evaluated the predictive value of AA diameter for RA plaque formation, with calculation of optimal cut-off values, sensitivity, and specificity, and the data were presented as mean±standard deviation. The statistical significance was set at p<0.05.

## RESULTS

The study included 130 patients. Demographic characteristics and morphometric measurements are presented in Table 1. The data sets were found to be normally distributed. When age-related changes were examined, it was seen that AA diameter and AA density values were significantly decreased in individuals over 65 years of age (p=0.025 and p<0.01, respectively). A significant difference was found between the genders in the mean right RA diameter (6.00±1.06 mm in women and 6.39±1.08 mm in men, p=0.048). No significant difference was found in the left RA diameter. The vertebral separation levels of the right and left RAs were examined, and the majority (right: n=49, left: n=46) were found to be separated between L1 and L2. The distribution of these levels showed a significant difference between genders (p>0.05). Gender- and age-related changes are summarized in Table 2.

**Table 1:** The demographic characteristics and morphometric measurements of the patients.

Sex		Age	AA diameter (mm)	Right RA diameter (mm)	Left RA diameter (mm)	Right RA length (mm)	Left RA length (mm)	Right RA separation angle	Left RA separation angle	AA density (HU)	Right RA density (HU)	Left RA density (HU)
Female	n	52	52	52	52	52	52	52	52	52	52	52
	Mean	65.52	20.77	6.00	7.20	57.98	49.07	58.62	67.90	314.89	278.33	286.27
	SD	14.96	5.48	1.06	8.96	16.33	17.54	17.40	16.83	108.05	108.30	109.27
	Median	66	20.05	6.06	5.76	60.45	52.20	55.50	67.05	313.50	265.70	288.40
	Minimum	25	12.20	3.30	4.00	14.94	4.62	.00	24.20	123.90	82.50	92.50
	Maximum	90	47.20	9.40	70.10	85.14	83.65	99.40	95.10	632.00	630.00	572.00
Male	N	78	78	78	78	78	78	78	78	78	78	78
	Mean	64.47	21.95	7.10	6.33	61.96	52.35	59.15	65.77	329.32	303.78	293.00
	SD	14.07	4.84	7.17	1.25	19.32	16.76	16.06	16.64	106.51	100.74	98.76
	Median	65.00	21.40	6.44	6.28	65.03	57.65	59.75	61.65	323.00	298.50	296.70
	Minimum	19	13.10	4.06	3.00	14.01	5.30	20.40	33.80	116.33	71.50	62.60
	Maximum	99	39.20	69.00	9.70	113.60	82.60	90.70	113.00	632.35	570.50	575.00
Total	n	130	130	130	130	130	130	130	130	130	130	130
	Mean	64.89	21.48	6.71	6.68	60.37	51.04	58.93	66.62	323.55	293.60	290.31
	SD	14.38	5.12	5.61	5.73	18.22	17.08	16.54	16.68	106.95	104.17	102.73
	Median	66.00	20.70	6.29	6.14	63.45	55.15	57.35	64.35	323.00	292.35	292.55
	Minimum	19	12.20	3.30	3.00	14.01	4.62	.00	24.20	116.33	71.50	62.60
	Maximum	99	47.20	69.00	70.10	113.60	83.65	99.40	113.00	632.35	630.00	575.00

AA: abdominal aorta; HU: hounsfield units; RA: renal artery; SD: standard deviation.

**Table 2:** Significant changes in the morphometric measurements depending on the sex and age.

	Age/sex	n	Mean	SD	p^*^
AA diameter	65-	67	22.45	4.22	0.025
	65+	63	20.44	5.79	
AA density	65-	67	357.38	114.69	<0.01
	65+	63	287.58	85.13	
Right RA diameter	Female	52	6.00	1.07	0.048
	Male	78	6.39	1.08	
Left RA diameter	Female	52	5.99	1.16	0.121
	Male	78	6.33	1.26	

*Independent sample t-test. AA: abdominal aorta, RA: renal artery, SD: standard deviation.

A moderate positive correlation was found between RAP formation and AA diameter (r=0.308, p<0.01). The ability of AA diameter to predict RAP formation was evaluated by ROC analysis. An AA diameter with a cut-off value of 20.5 mm was determined to be a significant risk threshold for plaque formation in RA (AUC: 0.708, sensitivity: 0.69, specificity: 0.68, p<0.01). Arteries with plaque were 6.97 mm longer than those without plaque (p=0.029). In patients with plaque formation detected in both AA and right RA, right RA length was associated with plaque formation (length difference: 10.55±3.53 mm, p=0.021). A significant relationship was observed between the increase in RA diameter (difference: 2.10±0.67 mm, p=0.017) and the enlargement of RA separation angle (difference: 27.44±10.04, p=0.042) and plaque formation in women (p<0.05).

In patients with plaque, the opaque material density values for AA and RA were significantly lower than in those without plaque. Patients with plaque in the left RA had a 2.71 mm larger AA diameter and 55.24 HU lower density in the same artery compared to those without plaque (p<0.01 for both). In patients with plaque in the right RA, the AA diameter was 3.84 mm larger (p<0.01), and the correct RA density value was 31.31 HU lower (p=0.048). The RA density values in patients with plaque formation were significantly lower than in those with only AA plaque formation and in those with no plaque formation. Still, this difference was not statistically significant (p>0.05).

## DISCUSSION

Our study examining the effect of RA morphometry on plaque formation indicates that anatomical factors play an essential role in the atherosclerotic process. Changes in RA diameter, RA length, and density were significantly associated with plaque formation. In our study, an AA diameter greater than 20.5 mm was identified as a significant risk factor for plaque formation in RA. A moderate positive correlation was found between plaque formation and AA diameter. Two studies have shown that increased atherosclerotic plaque in AA leads to vessel diameter expansion, which is associated with media thinning^12,13^. These findings suggest that the vessel diameter expansion observed in our study may not be solely a consequence of plaque but also a structural change that triggers plaque formation.

In addition to RA diameter, our study found that RA length also influences plaque formation. In patients with plaque, particularly in the right RA, the artery length was, on average, 6.97 mm longer than in those without plaque. According to hemodynamic principles, an increase in vessel length affects the pressure gradient in blood flow and, by creating variable shear stresses on the endothelial surface, may predispose to atherosclerotic plaque development^14^.

Our study found different results between the right and left RA. A negative correlation was found between plaque formation and density in the left RA. However, the same relationship did not reach statistical significance in the right RA. A similar lateralization difference was reported in a CT-based retrospective study by Yang and Yang^11^. This study demonstrated that plaque formation in the right RA was more strongly associated with age and hypertension^11^. In contrast, plaque formation in the left RA was more strongly associated with hypercholesterolemia. In our study, the majority of RAs were found to arise from the aorta at the L1–L2 vertebral level. Similar to literature studies, RAs generally arise from the aorta at the L1–L2 intervertebral disc space level^15^. In our study, the right RA diameter was significantly larger in men than in women. Conversely, a significant association was found between increased RA diameter and greater angle of separation in women, as well as with plaque formation. These findings are consistent with gender-related vascular differences reported in the literature. Bakker et al.^16^ demonstrated that men have more rupture-prone plaque characteristics, while women have a higher smooth muscle cell content. With age, the plaque composition in women becomes similar to that of men.

This difference is thought to be due to estrogen’s protective effect on the endothelium. Decreasing estrogen levels after menopause increase the risk of atherosclerosis^17^. In our study, the increased RA diameter in women increases the risk of plaque formation, which may be explained by the fact that increased vascular diameter impairs endothelial flow sensitivity and leads to endothelial dysfunction. In our study, we observed a significant decrease in AA diameter and density among individuals aged 65 and older. This decrease in density suggested that the contrast agent was not uniformly distributed throughout the vessel, resulting in reduced blood flow. Decreased cardiac output, increased arterial stiffness, and endothelial dysfunction associated with aging negatively impact renal perfusion and accelerate the development of atherosclerosis^18^.

The relationship between vascular structure and blood flow is critical to the pathogenesis of atherosclerosis. RA vessels are functional vessels that transport large volumes of blood per unit of time and have a large diameter relative to the organ volume. A living donor study by Kesavan et al.^19^ demonstrated a strong correlation between RA diameter and kidney volume. In our study, the increased RA diameter, particularly in women, triggered plaque formation, a finding contrary to expectations. When the vessel diameter expands, wall tension increases under constant pressure, according to Laplace’s law. However, if blood flow is not sufficiently increased, endothelial shear stress decreases, activating atherogenic processes^20^. The association between increased RA diameter and plaque formation in women in our study is consistent with this mechanism.

Our study highlights that RA plaque formation is influenced not only by traditional cardiovascular risk factors but also by anatomical and morphometric features. Topographic anatomy might sway the disease nature as well as prognosis^21,22,23,24,25^. Specifically, an AA diameter over 20.5 mm may serve as a clinical threshold for plaque risk. CT angiography-based morphometric measurements and RA density—both shown to reflect impaired renal perfusion even before plaque development—can aid early risk stratification. These parameters may offer valuable insights into renovascular disease progression, especially in patients with advanced age, hypertension, or diabetes, though further prospective validation is needed. Due to the retrospective nature of our study and incomplete patient records, key demographic and clinical risk factors (e.g., blood pressure, lipid profile, smoking status, BMI) could not be evaluated. Functional hemodynamic parameters were also not measured; our analysis focused solely on morphometric and density data. Future studies using Doppler ultrasonography or phase-contrast MRI may better clarify the link between morphometry, hemodynamics, and plaque formation.

## CONCLUSION

In essence, larger AA diameter and RA length are fundamental parameters affecting plaque formation. The density of opaque material in these areas may also be an essential criterion for assessing plaque risk in cases where plaque formation has not yet been observed.

## Data Availability

The datasets generated and/or analyzed during the current study are available from the corresponding author upon reasonable request.
